# Influence of Exercise Training on Skeletal Muscle Insulin Resistance in Aging: Spotlight on Muscle Ceramides

**DOI:** 10.3390/ijms21041514

**Published:** 2020-02-22

**Authors:** Paul T. Reidy, Ziad S. Mahmassani, Alec I. McKenzie, Jonathan J. Petrocelli, Scott A. Summers, Micah J. Drummond

**Affiliations:** 1 Department of Kinesiology and Health, Miami University, 420 S Oak St, Oxford, OH 45056, USA; reidypt@miamioh.edu; 2 Departments of Physical Therapy and Athletic Training, University of Utah, 520 Wakara Way, Salt Lake City, UT 84018, USA; Ziad.Mahmassani@health.utah.edu (Z.S.M.); alec.mckenzie@utah.edu (A.I.M.); jonathan.petrocelli@utah.edu (J.J.P.); 3 Department of Nutrition and Integrative Physiology, University of Utah, 250 1850 E, Salt Lake City, UT 84112, USA; scott.a.summers@health.utah.edu

**Keywords:** sphingolipids, fitness, obesity, insulin sensitivity, physical activity

## Abstract

Intramuscular lipid accumulation has been associated with insulin resistance (IR), aging, diabetes, dyslipidemia, and obesity. A substantial body of evidence has implicated ceramides, a sphingolipid intermediate, as potent antagonists of insulin action that drive insulin resistance. Indeed, genetic mouse studies that lower ceramides are potently insulin sensitizing. Surprisingly less is known about how physical activity (skeletal muscle contraction) regulates ceramides, especially in light that muscle contraction regulates insulin sensitivity. The purpose of this review is to critically evaluate studies (rodent and human) concerning the relationship between skeletal muscle ceramides and IR in response to increased physical activity. Our review of the literature indicates that chronic exercise reduces ceramide levels in individuals with obesity, diabetes, or hyperlipidemia. However, metabolically healthy individuals engaged in increased physical activity can improve insulin sensitivity independent of changes in skeletal muscle ceramide content. Herein we discuss these studies and provide context regarding the technical limitations (e.g., difficulty assessing the myriad ceramide species, the challenge of obtaining information on subcellular compartmentalization, and the paucity of flux measurements) and a lack of mechanistic studies that prevent a more sophisticated assessment of the ceramide pathway during increased contractile activity that lead to divergences in skeletal muscle insulin sensitivity.

## 1. Introduction

Obesity is a mounting health problem and the percentage of those who meet the classification is anticipated to double or triple in the next 40 years [1,2]. Obesity predisposes one to develop type 2 diabetes mellitus (T2DM), the prevalence of which is also expanding at an alarming rate [3]. Metabolic impairments associated with obesity increase the risk of developing other troubling health conditions (e.g., cardiovascular disease, peripheral vascular disease, and neuropathy), leading to enormous healthcare cost and decreased quality of life [3,4]. The hallmarks of obesity and T2DM, glucose intolerance and insulin resistance, can be observed before their respective diagnoses during a state of pre-diabetes.

Of those suffering from these metabolic derangements, older adults make the largest proportion and as of 2017 ~48% of older adults are considered pre-diabetic [5]. Since insufficient physical activity is one of the top 10 leading causes of mortality across the globe [6] it is no surprise that inactivity is also one of the primary causes of metabolic dysfunction [7,8] and that many of the aging-related declines in health are related to low physical activity levels [9,10,11]. It is now well understood that older adults have the highest prevalence of inactivity both in the United States [12] and across the globe [6]. Physical activity is arguably the most comprehensive prevention and treatment for obesity [11] and its metabolic comorbidities [7,8,13,14]. Interestingly, the insulin resistance (IR) associated with aging may share a similar mechanism to that of IR that accompanies physical inactivity [15,16,17,18], further indicating the linkage between aging and physical inactivity.

The focus of this review is to examine the effects of physical activity in aging on a class of lipid intermediates, ceramides, that have been implicated in obesity and inflammation-induced skeletal muscle insulin resistance and impair insulin signaling downstream of the receptor [19].

### 1.1. Skeletal Muscle Ceramides and Physical Inactivity-Induced Insulin Resistance

Can ceramides, a dominant mechanism of diet-induced IR, also be a viable candidate by which physical activity (exercise) induces major swings in insulin sensitivity? To address this question, we evaluated the literature comprising rodent and human investigations of chronic exercise in the context of aging. We considered whether the robust changes in insulin sensitivity tied to exercise habituation correspond with the levels of skeletal muscle ceramides, as well as with the expression or activity of enzymes governing ceramide metabolism. Although, we will discuss all phenotypes, for the purpose of this review, our primary focus was on healthy phenotypes (non-obese, non-diabetic adults), across the lifespan in order to remove the confounding effects of current metabolic dysfunction and understand the extent to which the pathways related to overnutrition-induced muscle dysfunction relate to the pathways resulting from physical activity.

A hallmark of diabetes is obesity and the ectopic deposit of fat in many tissues, including skeletal muscle [20]. An overabundance of intramuscular lipids leads to an accumulation of lipid intermediates (diacylglycerol (DAG), acetylcarnitines, and ceramides) as a mass action effect, with some of these lipid intermediates worsening insulin sensitivity by inhibiting insulin signaling [21]. In addition, obesity-induced redox stress and inflammation intensify the metabolic dysfunction [22]. Clever genetic interventions in rodent models indicate with certainty that ceramides drive obesity-induced IR in pre-clinical models [19,23], but their role in human insulin resistance has been controversial [24]. Additionally, aging skeletal muscle has been associated with greater skeletal muscle ceramide in some cases [25,26,27], but not others [28,29] and it is unclear if this is the result of aging per se or due to a progressive decline in physical activity levels over the years. The dilemma is exacerbated when examining models of physical activity, which has received far less attention, and no studies using genetic models to pinpoint mechanisms.

### 1.2. Source of Substrate for Production and/or Accumulation of Ceramide in Muscle

Ceramide production relies on the availability of fatty acids and serine, and the overabundance of these nutrient precursors drives accumulation of the sphingolipid in models of overnutrition [20,21,30]. Not all dietary fats are considered equal, as ceramides are highly dependent upon the presence of saturated fatty acids such as palmitate. Of note, exercise stimulates lipolysis to provide free fatty acids (FFAs) as energy for contracting muscles. Ceramide content in various tissues may also be regulated through tissue export and import to and from different tissue types. For example, the liver can export ceramides on very low density lipoproteins (v-LDL) for delivery to muscle [31]. Additionally, sphingoid bases can be exported from muscle into the circulation [32,33].

The diversity of ceramide species is a reflection of the activity of the six ceramide synthase (CERS) enzyme isoforms, which differ by tissue distribution and substrate specificity (for more detailed review see [34]). CERS1, 4, 5 and 6 have the highest expression in skeletal muscle thus their C-16:0 and C-18:0 ceramide products are the most abundant [34] in the tissue. Coincidentally, these species are also most commonly related to IR [35,36,37], specifically in human skeletal muscle [37,38,39,40,41].

### 1.3. Intracellular Biochemical Pathways and Enzymes Controlling Ceramides

Of the thousands of sphingolipids present in cells, ceramides are a particularly intriguing class of molecules. In addition to serving as common precursors for all other highly abundant sphingolipids, they have distinct bioactivities in cellular stress responses [42]. Ceramide production occurs through three pathways: de novo synthesis from fatty acids and serine; a salvage pathway that occurs through its degradation and re-formation in lysosomes; or sphingomyelin hydrolysis [42].

De novo biosynthesis of ceramide starts with the rate limiting condensation of palmitoyl-CoA with L-serine through serine palmitoyltransferase (SPT), producing the transient intermediate 3-ketosphinganine. 3-ketosphinganine is then reduced to sphinganine by 3-ketosphinganine reductase. The (dihydro)ceramide synthase (CerS 1–6) enzymes acylate sphinganine to generate dihydroceramide, which is further modified by dihydroceramide desaturase (DEGS 1–2) through the placement of a 4,5-*trans*-double bond. At this point ceramide can be metabolized to (1) glycosphingolipids through glucosylceramide synthase, (2) ceramide-1 phosphate with a ceramide kinase, or (3) sphingomyelin through sphingomyelin synthases.

The salvage and sphingomyelinase pathways also influence ceramide levels. Hydrolysis of the far more abundant sphingomyelin can occur in plasma membranes or intracellular organelles. The various SMases, including neutral Mg^++^ dependent sphingomyelinase (nSMase) or acid sphingomyelinase (aSMase), transfer the choline residue of SM to DAG to generate phosphatidylcholine. The rate of ceramide degradation is also controlled by three isoforms of ceramidase: acid (aCDase), neutral (nCDase) and alkaline (alCDase). These enzymes hydrolyze the lipid to generate sphingosine and a FFA. Recent studies have found that adiponectin receptors are another class of ceramidases, degrading the lipid in response to the binding of the insulin-sensitizing adipokine [43,44,45,46]. The sphingosine produced from these ceramidase reactions can, in turn, be phosphorylated to produce sphingosine-1-phosphate (S1P), which has its own set of biological actions, often opposing those of ceramides.

Theoretically, ceramide accumulation in skeletal muscle could arise through several potential routes: (1) increased availability of substrate for ceramide biosynthesis through mass increases in circulating FFA [47]; (2) reduced palmitate oxidation and increased partitioning of palmitate [48]; (3) increased ceramide de novo synthesis via activation of toll-like receptor 4 (TLR4) [49,50] or tumor necrosis factor alpha (TNFα) [51]; (4) decreased ceramide turnover [37]; (5) increased sphingomyelinase activity; (6) increased serum delivery via circulating low density lipoprotein (LDL) [52]; (7) reduced ceramide clearance; or (8) a combination of the above. These factors could be predicted to promote ceramide abundance as a result of inactivity to potentially promote muscle IR. However, most of these routes have not been investigated in the context of physical activity in humans or rodents.

## 2. Aging and Ceramide

Skeletal muscle IR is a common feature of aging [15,53], but it has yet to be determined if this metabolic defect is the result of biological aging, ceramides or influenced by the lifestyle changes common in aging (Table 1). For example, two independent cross-sectional studies have demonstrated that activity levels and obesity (rather than aging) influence muscle insulin sensitivity [54,55], but ceramides were not measured. Aging skeletal muscle has been associated with greater skeletal muscle ceramide in some cases [25,26,27], but not others [28,29]. The reasons for these discrepancies may stem from the confounding influence of obesity in aging and differences in the methods to assay ceramides. In the cases were ceramides were more abundant in aging, the older adults were obese [25,26,27], but ceramides were similar [29] or even greater in the young [28] when examining leaner age groups. It is interesting to note that greater fitness levels appear to disassociate the relationship between increased age and muscle ceramide content and potentially with insulin sensitivity. In their first study, Søgaard et al. found greater content of ceramides, including the C:16 and C:18 species in the young compared to the old participants [28], but this may be due to the lean status or greater fitness level (VO_2_peak: 30–40 mL∙kg^−1^∙min^−1^) in both the old and young groups compared to the cohort in their next report demonstrating a greater content with aging [25]. In that report their cohort of old and young participants was obese and had a lower fitness level (VO_2_peak: 20–30 mL∙kg^−1^∙min^−1^) [25]. Future investigations need to isolate the effect of body composition, fitness level and aging on skeletal muscle ceramides.

## 3. Longitudinal Studies in Rodents

The literature on exercise training and muscle ceramide content in rodent is varied (Table 2). The first longitudinal exercise training study (six-weeks forced treadmill running) by Dobrzyn et al. showed decreased ceramide content in the soleus and red and white gastrocnemius of male rats [60]. Similar, but less pronounced effects were observed with muscle sphingomyelin with slight differences between muscle groups. The same study reported that exercise increased muscle sphinganine and nSMase, but not sphingosine, and suggested that ceramide flux through de novo synthesis rather than the salvage pathway may have been altered with training [60]. In contrast, a study examining 8-weeks of voluntary wheel running in male rats, did not reveal any changes in gastrocnemius lipids (phosphatidyl choline, phosphatidyl ethanolamine, phosphatidyl inositol, phosphatidyl serine, cardiolipin, ceramides, sphingomyelin, and lysophosphatidyl choline) except for phosphatidyl inositol, which decreased [57]. The divergent results of this current study compared to Dobrzyn et al. [60] may have been due to the form of exercise (forced treadmill running vs. voluntary wheel running), limited duration of treatment or more likely that the rats from the Dobrzyn et al. study were larger and older.

A following study from Dr. Jan Gorski’s group using a more robust form of endurance exercise in male rats (i.e., treadmill running) demonstrated increased insulin sensitivity (HOMA-IR) and sphinganine in the soleus and red gastrocnemius similar to the previous report [60] but no change in sphingosine or S1P, dihydroceramide or ceramide [58]. They also showed an increase in SPT2 and aSMase activity (in the soleus) but only an increase in sphinganine-1-phosphate content and nSMase activity isolated to the gastrocnemius. [58]. Interestingly, enzymes related to the degradation of ceramide to sphingosine, nCDase and alCDase, were decreased, yet ceramide content was unaltered. The authors speculated that other pathways of ceramide turnover (ceramide kinase, sphingomyelin synthase or glucosylceramide synthase) could have offset changes in ceramide abundance with exercise training. However, Hollaway and colleagues used chronic electrical stimulation to the hindlimb muscles (6 h/d for 6 d) in lean and obese female (Zucker) rats, which resulted in improved insulin-stimulated glucose transport to muscle and increased TAG content and composition [59]. These authors also demonstrated an increase in the size and number of lipid droplets in the subsarcolemal and intramyofibrillar regions with exercise training as reviewed elsewhere [61]. Ceramide content and composition (species-specific) only decreased in obese, but not the lean rats [59]. These data provide further credence to the concept that chronic muscle contraction reduces the level of lipid intermediates only in conditions of frank IR associated with dyslipidemia, such as obesity and diabetes. Increased contractile activity increases long chain fatty acids into muscle as they are the necessary substrate to fuel chronic activity [62]. This increased transport of lipids into muscle is similar to overfeeding, however chronic activity increases oxidation of lipids and prioritizes lipid storage to reduce accumulation of lipid intermediates [61]. In fact, palmitate and FFA transport is increased with contractile activity [62,63] and decreased during denervation (inactivity), a phenomenon that correlates with the abundance of membrane bound fatty acid transport proteins [62]. Thus, chronic exercise may reduce lipid intermediates, such as ceramide, only if they are profoundly elevated due to dietary excess or genetic disposition. Also, this discussion bring up the point that slow-twitch myofibers contain greater amounts of ceramide [64] and rodents typically have more fast-twitch muscle than humans, a factor that may explain some of the complexity and variation in the field.

## 4. Cross-Sectional Studies in Humans

When examining cross-sectional studies of exercise-trained individuals compared to those that are sedentary, results when evaluating muscle ceramides are fairly ambiguous (Table 3 and Supplementary Table S1). The variation might be explained by body mass index (BMI) status rather than physical activity level, since ceramides are linked to IR in extremes of body mass [66]. Of note, cross-sectional studies between active and sedentary groups involve more than just physical activity level (e.g., genetic predisposition and daily nutritional intake), and that several of these studies are potentially underpowered [23] thus introducing a large source of variability. Nonetheless the use of age and BMI-matched sedentary controls with exercise-trained participants is a powerful design strategy to address the initial question of whether physical activity level and insulin sensitivity [75] are related to skeletal muscle ceramides. In one study, examination of healthy age- and BMI-matched exercise-trained and sedentary young adults found no difference in skeletal muscle ceramide content or species at rest, but less sphingomyelin was found in exercise-trained participants [65]. Another more recent study demonstrated that among relatively lean young and older participants there was no difference in skeletal muscle ceramide based on training status [28]. In contrast, another study showed greater basal total ceramide content and insulin sensitivity in trained vs. sedentary BMI-matched young subjects [68]. A possible explanation for these seemingly divergent findings could be that untrained participants in the former study were of similar aerobic fitness (see VO_2_ peak data in Table 3) to the trained subjects from the later study. Another consideration is that the greater ceramide content in the more trained subjects [68] may be indicative of active lipid delivery in trained individuals, and not necessarily a predisposition to cause IR. Further, a well-powered cross-sectional comparison of endurance-trained, sedentary and obese older adults demonstrated pronounced stepwise decreases in insulin sensitivity, yet only the obese group had elevated skeletal muscle ceramide content [67]. In that study, the difference in insulin sensitivity between the sedentary and endurance trained older adults was ~20% and the two groups showed identical ceramide content and composition [67] suggesting a discordant relationship between insulin sensitivity and ceramides in the absence of obesity. This pattern was also shown in a cross-sectional comparison of exercise-trained and sedentary middle-aged adults with distinctly different insulin sensitivity levels [66]. A recent in-depth cross-sectional investigation by the laboratory of Dr. Brian Bergman compared middle-aged athletes, lean controls, obese controls, and T2DM patients to demonstrate similar stepwise and distinct group differences in insulin sensitivity and non-oxidative glucose disposal [38]. There were no differences in skeletal muscle total, mitochondrial/ER, nuclear, sarcolemmal, or cytosolic ceramide content and composition between age- and BMI-matched athletes vs. lean controls [38]. Sub-cellular ceramide localization of C18:0 and insulin sensitivity were correlated with pooled data from all the participants, however this relationship appeared to be driven by inclusion of the group of persons with T2DM [38] and independent of the physical activity level between athletes and lean controls. In summary, cross-sectional studies in young and old adult humans demonstrate that levels of insulin sensitivity due physical activity status are unlikely to be explained by skeletal muscle ceramide content or ceramide species. By comparison, these data indicate that muscle ceramides are likely to be elevated and related to insulin sensitivity during metabolic disturbances such as obesity and T2DM.

## 5. Longitudinal Studies in Humans

The improved insulin sensitivity following exercise training is well established [75]. Longitudinal exercise training studies (>8-weeks) increase insulin sensitivity [76,77,86] and this occurs while decreasing IMTG content in T2DM [76] but not in otherwise healthy obese [76,77,86] humans. Alternately, IMTG has been shown to increase with exercise training in obese older adults coupled with a decrease in muscle ceramide content [78,79]. This might suggest a partitioning of triacylglycerol (TAG) and IMTG to specific cellular locations after chronic exercise training [80,81]. In these studies, exercise training shifted IMTG storage away from the sarcolemmal fraction and in closer proximity to the mitochondrial and myofibrils–theoretically for more efficient access of fuel for use in contraction [61,80,81]. Interestingly, the storage of C18:0 ceramide in the sarcolemmal fraction of the cell is most associated with IR [38]. One could speculate that with physical inactivity the ectopic lipid storage in muscle may likely occur in the sarcolemmal regions of the cell where inhibition of insulin signaling is most likely to occur but this remains to be examined. Although no differences in cross-sectional studies between lean athletes and lean sedentary controls exist regarding the content, composition or localization of skeletal muscle ceramide [87] what remains unknown is if the cellular distribution of ceramide changes after a period of exercise training or physical inactivity to reflect the associated changes in insulin sensitivity.

Skeletal muscle ceramides decrease in obese [77,78,82,83,86] and T2DM [83] humans following aerobic exercise training or aerobic interval training [25,86] (Table 4 and Supplementary Table S2). However, exercise training studies in participants with a wide range of insulin sensitivities, including normal weight controls [84], normal weight offspring of T2DM [84] and lean men [81] and women [80] largely result in no changes in skeletal muscle ceramide content or specific ceramide species following similar aerobic exercise training. One recent study had young and old obese participants complete 6 weeks of aerobic interval training and demonstrated that only the older participants decreased select ceramide species [25], this was likely driven by a greater initial ceramide content in those older but obese participants. Eight of the ten training studies reported ceramide composition (i.e., specific moieties) and the two studies in lean subjects that reported no change of ceramides used a crude DAG kinase assay to assess ceramide content [80,81]. This latter assay does not distinguish ceramides from dihydroceramides nor does it resolve ceramides of differing chain lengths. Even though total ceramide was not changed in normal weight controls and offspring of T2DM, exercise training decreased C22:0, yet this was not associated with the improvement of insulin sensitivity [84]. A few other studies in obese subjects depicted a change in several other species after aerobic exercise training [76,82] or interval training with ceramide C18:0 [86], however these changes were also not related to insulin sensitivity. A factor for consideration is that some [77,81,85,86], but not all [82,84] of these studies were underpowered. Alternatively, exercise training studies in obese older adults [78,79] or obese adults with T2DM [83] display negative associations with total [78,79,83], C14:0 [83], and C-16:0 or C-24:1 [79] ceramide and insulin sensitivity. However, the mechanism (i.e., de novo, salvage, hydrolysis) for exercise training-induced changes in ceramides and enzymes of ceramide metabolism in human skeletal muscle is vastly unexplored. A need exists for a more comprehensive examination of skeletal muscle ceramide at the level of specific enzymes of ceramide metabolism and most importantly with the recently developed flux assays [40,88,89]. Interestingly, ceramide metabolism may influence other functions, such as exercise performance [90].

In summary, changes in ceramide content after exercise training may occur in individuals with obesity or T2DM, likely do not change in healthy individuals and these factors drive the impact of age. These improvements following exercise training in metabolically compromised individuals may occasionally be associated with the improved insulin sensitivity following exercise training. A major limitation appears to be the reliance on whole cell lysate ceramide content/composition, which may not be as precise as studies assessing subcellular localization or ceramide flux.

## 6. Mechanisms and Considerations

### 6.1. Are Ceramides Involved in the Development of IR or a Mechanism to Worsen the Severity of Inactivity-Induced IR in Aging?

A panoply of studies with rodents clearly identifies a role for skeletal muscle ceramides in mediating insulin sensitivity under conditions with pronounced derangements in metabolic function [23,87,91]. Yet, even under extreme differences in metabolic function the association of skeletal muscle ceramide content with IR in humans is inconsistent [24,66,92] and not well correlated [93]. Ceramides are not linked to skeletal muscle IR in aging specifically, but only with extremes of body mass [66], severity of metabolic dysfunction linked to diet [94], or disease [21,37,92,95]. Collectively, these reports suggest that skeletal muscle ceramide content may not be involved in the development of IR from physical inactivity from aging, though they likely worsen the condition during aging-associated obesity and metabolic dysfunction.

### 6.2. Subcellular Assessments of Ceramide

If the effect of ceramide on IR in healthy humans is subtle then the assessment of ceramide content and composition needs to be specific to capture the relationship. More recent reports have revealed that skeletal muscle subsarcolemma C16:0 [40,89] and sarcolemmal or mitochondrial or nuclear C18:0 [38] ceramide are more tightly associated with insulin sensitivity. This is an important point since the majority of studies evaluated in this review article assess the total skeletal muscle homogenate while some measured the specific ceramide species and only two studies to date have examined the subcellular localization of muscle ceramide [38,64]. Whether physical inactivity-induced IR is modulated through a certain lipid intermediate species in a specific myocellular location remains sparsely examined [64]. Exercise training shifts intramuscular triglycerides from subsarcolemma to intramyofibrillar and mitochondrial locations [61,80] to more efficiently store fuel for contraction [61]. Therefore, it would be intriguing to extensively test the hypothesis that during the time course of inactivity there is a gradual shift in lipotoxic ceramides to the subsarcolemal region where their presence is more likely to inhibit insulin signaling. Indeed, the localization of ceramide or diacylglycerol near the plasma membrane is a key determinate of the signaling actions to inhibit insulin action. Muscle cell culture models from divergent species demonstrate differences in the mechanism of ceramide to inhibit insulin signaling in culture [96] and in vivo [97]. Subcellular localization to the sarcolemmal region is likely a mechanism where ceramide can inhibit various portions of the insulin signaling cascade [98]. Saturated ceramides are more prone to alter membrane lipid rafts and are thus able to manipulate cellular function [99], such as protein and various receptor actions [100]. Ceramides, such as C18:0, are also known to have more direct actions to inhibit insulin signaling via protein phosphatase 2 (PP2A) [101]. In addition to plasma membrane localization to inhibit insulin signaling, accumulation of ceramide in other areas of the cell (mitochondrial, nuclear) may be involved [38] in the dysregulation of muscle metabolism. When considering muscle ceramides, one cannot exclude the effects of other non-muscle cell types residing in muscle that could contribute to the ceramide pool. Immune [102], and endothelial cells are potential sources of ceramide in skeletal muscle and as such it is difficult to differentiate the sources of ceramide found in skeletal muscle homogenate or even the subcellular locations.

### 6.3. Conclusions

Altogether, this review of the literature points to a dynamic response of skeletal muscle ceramide metabolism to physical activity, the significance of which is not fully understood. Aging itself does not appear to impact skeletal muscle ceramide content independent of physical inactivity, obesity and metabolic disruption that typically are associated with the aging process. Cross-sectional comparison participants by activity level and chronic exercise training does not support a relationship between skeletal muscle ceramide content and insulin sensitivity in healthy individuals. In agreement with this finding, chronic exercise training does not consistently reduce ceramide content linked to improved insulin sensitivity—a relationship that is occasionally observed in participants with robust metabolic dysfunction. Future considerations include more frequent assessments and longer durations of physical activity and inclusion of sex as a biological variable. Thus, the role of ceramide lipotoxicity in the early development of aging-induced IR is yet to be determined and resolution of this question would greatly benefit from the modern comprehensive lipidomic assessments of sphingolipids, in specific myocellular localizations and certainly with addition of ceramide flux assays. The possibility of subtle subcellular redistribution of skeletal muscle ceramide not detectable in whole muscle homogenate cannot yet be discounted in the etiology of age-induced skeletal muscle IR. If skeletal muscle ceramide intramyocelluar distribution and content is not involved in the development of age-induced IR, it is probable that ceramides may serve as a likely mechanism to worsen the progression of metabolic dysfunction into metabolic disease during longer durations of inactivity and during aging.

### 6.4. Future Challenges and Directions

Clearly, a divergence in the rodent [23,103] and human research [24] is present surrounding the effects of skeletal muscle ceramide on metabolism. These observations could suggest (1) intrinsic differences between rodents and humans surrounding overall metabolism and ceramide content/composition [65], (2) differences in the analytical methods to quantify ceramides [91], (3) imprecise and non-biologically specific assessment of lipid intermediates to capture modest differences (compared to rodent research) in insulin sensitivity and (4) the reality that we can only sample a small percentage of human muscle whereas the whole muscle or a larger portion is sampled from the rodent. Even with these limitations, there is a need to conduct mechanistic mouse studies of ceramide metabolism examining the interaction between aging, physical activity level and insulin sensitivity.

## 7. Summary

- Though lipid/obesity induced insulin resistance is well examined, the mechanism(s) linking activity to insulin sensitivity is largely unknown, particularly in aging.
- Aging is only linked to increased skeletal muscle ceramide content in obese individuals.
- Cross-sectional studies of exercise-trained adults reveal a range of insulin sensitivities related to physical activity level that are independent of skeletal muscle ceramide content and species. Indeed, acute exercise in healthy humans may actually increase ceramide content within skeletal muscle.
- By comparison, longitudinal studies indicate that exercise may not impact ceramides in skeletal muscle of healthy individuals but is rather more likely to decrease skeletal muscle ceramides in individuals with previously elevated ceramides resulting from obesity or T2D.
- Clarity of the role of skeletal muscle ceramides on insulin sensitivity during physical activity may be enhanced with improved methodologies to evaluate distinct ceramide species (e.g., with different acyl-chains), their subcellular distribution, and their turnover. Additionally, there is a need for mechanistic genetic studies of ceramide metabolism examining the interaction between physical activity level and insulin sensitivity. Greater attention to these nuances of ceramide action could unveil relationships which may be masked in prior literature.

## Figures and Tables

**Table 1:** Cross-sectional studies in aging humans at baseline.

Reference	Subjects	Age (y)	Aerobic Fitness	Health/BMI	Ceramide Method	Muscle Ceramide	Ceramide Species	IS Method	IS	Associations with IS	Other
Rivas et al. (2012) [26]	Young (9 M)	22 ± 1	-	Lean	HPLC-MS	-	-	-	-	None examined	C:16 - associated with leg lean mass and strength
	Old (10 M)	74 ± 2	-	OW		= Young	> C:16, C:20 and Sat vs. Young	-	-		
Moro et al. (2009) [27]	Lean (10 M/6 F)	24 ± 1	-	Lean	LC/ESI/MS/MS			HE Clamp ^	8.3 ± 0.7	Ceramides not associated with age	IMTG + correlated with ceramides
	Obese (14 M/18 F)	41 ± 3	-	Obese		> vs. Lean Young	> Sat vs. Lean Young		6.1 ± 0.4		
Søgaard et al. (2019) [28]	Young (11 M)	23 ± 1	VO_2_peak-Rel: 46 ± 1 *	Lean	TLC-HPLC	622 ± 74	> C:16, C:18, C:22 vs. both OLD	Insulin 120-min OGTT (pmol·L^−1^)	87.9 ± 14.8	In young: No association in the old	-
	Old (18 M)	66 ± 1	VO_2_peak-Rel: 31 ± 1 *	Lean/OW		410 ± 66	-		97.6 ± 55.3		-
Chee et al. 2016 [29]	Young Lean (*n* = 7)	21y ± 1	VO2peak-Rel: 57 ± 2 ‡	Lean	LC/ESI/MS/MS	-	-	HE Clamp, (mg·kg LBM^−1^·min^−1^) & skeletal muscle 2-DG accumulation	65 ± 6.0	Only with overweight	-
	Old Lean (*n* = 7)	70 ± 1	VO_2_peak-Rel: 45 ± 2 ‡	Lean		-	-		58 ± 6		-
	Old Overweight (*n* = 7)	69 ± 1	VO_2_peak-Rel: 40 ± 2 ‡	OW		-	C:20: > Young Lean		42 ± 5		largely driven by differences in BW
Søgaard et al. (2019) [25]	Young (5 M/9 F)	32 ± 2	VO_2_max-Rel: 28.3 ± 1.2	Obese	LC/ESI/MS/MS	-	-	HOMA-IR	2.14 ± 0.24	Not reported	-
	Old (11 M/11 F)	63 ± 1	VO_2_max-Rel: 25.2 ± 1.0	Obese		Sat, C:16, C:18, C:18:1: > Young Obese	Sat, C:16, C:18, C:18:1: > Young Obese		1.88 0.23		

>, greater than; BMI, Body Mass Index; BW, Body weight; Cer, Ceramides; F, female; HE, hyperinsulinemic euglycemic; HOMA-IR, Homeostatic Model Assessment of Insulin Resistance, IMTG, intramuscular trigycerides; IS, insulin sensitivity; M, male; min, minutes; *n*, number of subjects; OW, overweight; Sat, saturated; VO_2_peak, peak oxygen uptake; y, years; ^, μmol glucose infused·kg FFM^−1^·min; Rel, relative * (milliliters per kilogram per minute ); ‡, (milliliters per kilogram of fat free mass per minute ).

**Table 2:** Exercise training studies in rodents.

Reference	Subjects	Age	Aerobic Fitness	Health/BMI	PA Modification	Ceramide Method	Muscle Ceramide	Ceramide Species	IS Method	IS (PRE)	IS (Post)	Associations	Note
Dobrzyń et al. (2004) [56]	Rat (Wistar) (10 M)	250–280 g	Sed	Lean	Control	TLC, GLC	~135 nmol/g	-	-	-	-	-	-
	Rat (Wistar) AET (10 M)	250–280 g	Sed	Lean	6 wk forced treadmill running		~80 nmol/g ↓, sat in oxidative muscles	↓ C14, C16, C16:1, C18, 20:4, 22, 24:1	-	-	-	-	↓ SM, ↑ sphinganine nSMase, ↔ sphingosine
Tsalouhidou et al. (2009) [57]	Rat (Wistar) Sed (15 M)	7 wk	Sed	Lean	Control	one-dimensional TLC-GC	0.33 ± 0.16 µmol/g	↔	-	-	-	None	PC, PE, PI, PS, CL, SM and LPC unchanged
	Rat (Wistar) AET (20 M)	7 wk	Sed, Improved	Lean	8 wk voluntary wheel running		BL=, 0.27 ± 0.06 µmol/g	↔	-	-	-		31% ↓ PI
Błachnio-Zabielska et al. (2011) [58]	Rat (Wistar) Sed (8 M)	200–250 g	Sed	Lean	Control	HPLC (C17 std)	24.57 ± 3.53 nmol/g wet wt	-	HOMA-IR	-	1.13 ± 0.10	Wrong direction	-
	Rat (Wistar) AET (8 M)	24 h post	Sed	Lean	5 wk forced treadmill running		28.70 ± 3.59 nmol/g wet wt	-	HOMA-IR	-	0.54 ± 0.04 ↓	Wrong direction	Plasma FFA >100% lower, > Sphinganine, SPA1P, SPT2 and aSMase. ↓ nCDase and alCDase
Holloway et al. (2014) [59]	Rat (Zucker) (18 F)	~255 g	Sed	Lean	6 d e-stim 6 h/d	TLC, GLC	↔ Red, ↓ White	↔ Red, ↓ White	3-OMG in perfused hindlimb	Lean > Obese	↑, but Lean > Obese	+ TAG, lipid droplet size	↑ lipid droplets
	Rat (Zucker) (18 F)	~350 g	Sed	Obese	6 d e-stim 6 h/d		↓↓	↓↓ C18		-	↑	+ TAG, lipid droplet size	↑↑ lipid droplets

↑, increase; ↑↑, large increase; ↓, decrease; ↓↓, large decrease; ↔, no change; 3-OMG, 3-O-methylglucose; AET, aerobic exercise training; alCDase, alkaline ceramidase; aSMase, acid sphingomyelinase; BMI, Body Mass Index; BL, baseline; CL, cardiolipin; d, days; e-stim, electrical stimulation; F, female; FFA, free fatty acids; g, grams; GC, gas chromatography; GLC, gas–liquid chromatography; h, hours; HOMA-IR, homeostatic model assessment of insulin resistance; HPLC, high-performance liquid chromatography; IS, insulin sensitivity; LPC, lysophosphatidyl choline; M, male; *n*, number of subjects; nCDase, neutral ceramidase; nmol/g, nanomole per gram; nSMase, neutral sphingomyelinase; PC, phosphatidyl choline; PE, phosphatidyl ethanolamine; PI, phosphatidyl inositol; PS, Phosphatidyl serine; Red, red portion of gastrocnemius; sat, saturated; Sed, sedentary; SM, sphingomyelin; SPA1P, sphinganine-1-phosphate; SPT2, serine palmitoyltransferase 2; TAG, triglycerides; TLC, thin-layer chromatography; White, white portion of gastrocnemius; wk, weeks; wt, weight.

**Table 3:** Cross-sectional studies and muscle ceramides in humans.

Reference	Subjects	Age (y)	Aerobic Fitness	Health/BMI	Muscle Ceramide	Ceramide Species	IMTG	Associations with IS
Helge et al. (2004) [65]	Untrained (*n* = 8)	26 ± 1	VO_2_peak-, Rel: 50.8 *	Lean	~200 nmol/g	No difference between groups	-	-
	Trained (*n* = 8)	28 ± 2	VO_2_peak-, Rel: 62.5 *	Lean	~200 nmol/g		-	-
Skovbro et al. (2008) [66]	T2D (*n* = 8)	54 ± 2	VO_2_peak-Rel: 31 ± 3 *	T2D/Obese	108 ± 7 nmol/g	Trained > IGT	68.9 ± 21.4 nmol/mg	(*r* = 0.42, *p* < 0.05) with IS and muscle Cer
	IGT (*n* = 9)	54 ± 2	VO_2_peak-Rel: 37 ± 2 *	IGT/Obese	95 ± 6 nmol/g		38.5 ± 6.8 nmol/mg	
	Controls (*n* = 8)	53 ± 2	VO_2_peak-Rel: 43 ± 2 *	OW	126 ± 12 nmol/g		35.6 ± 10.0 nmol/mg	
	Trained (*n* = 8)	51 ± 2	VO_2_peak-Rel: 58 ± 2 *	Lean	156 ± 25 nmol/g		49.7 ± 12.6 nmol/mg	
Amati et al. (2011) [67]	Obese (11 M/10 F)	67 ± 1	VO_2_peak-, Rel: 33 *	Obese, pre-diabetes	160 ± 18 nmol/g	Obese >, Athletes, NW sed (C18:1, 24:0, 24:1)	Ath, Obese > NW sed for content and droplet density	Total DAGs (*r* = 0.57, *p* = 0.05), total Cer (*r* = −0.48, *p* < 0.05)
	NW (3 M/4 F)	67 ± 2	VO_2_peak-, Rel: 42 *	NW	80 ± 27 nmol/g			
	Athletes (10 M/4 F)	65 ± 1	VO_2_peak-, Rel: 53 *	Lean/NW	83 ± 21 nmol/g			
Chow et al. (2014) [68]	Trained (8 M/7 F)	24 ± 1	VO_2_peak-Rel: 49 ± 2 *	Lean	42.6 ± 4.6 ng/mg	↑ sat	4.0 ± 0.5 μg/mg	No
	Sed (7 M/6 F)	2 w ± 0.6	VO_2_peak-Rel: 39 ± 1 *	Lean	30.3 ± 2.7 ng/mg		3.9 ± 0.5 μg/mg	
Chow et al. (2017) [69]	Trained (16M/13F)	26 ± 0.9	VO_2_peak-Rel: 62–71 ‡	Lean	-	-	T1: ↑↑	-
	Sed (15 M/13 F)	23 ± 0.6	VO_2_peak-Rel: 53–56 ‡	Lean	-	-	T1: ↑	-
Bergman et al. (2010) [70]	Athletes (*n* = 11)	23 ± 0.7	VO_2_peak-Rel: 68 ± 2 *	Lean	-	-	~21 = IMTG saturation,	DAG% saturation (curvilinear)
	Controls (*n* = 11)	21 ± 0.7	self-reported < 2 h PA per wk	Lean	-	-	>16:0, 16:1, <18:0, 18:2	
Baranowski et al. (2011) [71]	Sed (*n* = 10)	20 ± 0.7	VO_2_peak-Rel: 47 ± 3 *	Lean	Plasma 62.4 ± 16.4 < in RBCs	-	-	-
	Trained (*n* = 10)	21 ± 0.9	VO_2_peak-Rel: 57 ± 6 *	Lean	Plasma 60.8 ± 11.1	-	-	-
Bergman et al. (2012) [72]	Obese (4 M/2 F)	40 ± 2	VO_2_peak-Rel: 25 ± 4 *	Obese	-	-	-	total and Sat DAG in skeletal muscle membranes
	T2D (6 M)	44 ± 2	VO_2_peak-Rel: 25 ± 2 *	OW/ Obese-T2D	-	-	-	
	Athletes (8 M/2 F)	35 ± 3	VO_2_peak-Rel: 56 ± 5 *	lean	-	-	-	
Bergman et al. (2015, 2016, 2018) [37,73,74]	Athletes (11 M/4 F)	41 ± 1	VO_2_peak-Rel: 48 ± 4 *	Lean	Serum: Not different in obese	-	-	C16:0 Cer and C18:0 sphingomyelin correlated w/ whole body insulin resistance
	T2D (11 M/4 F)	43 ± 1	VO_2_peak-Rel: 19 ± 3 *	Obese-T2D	Serum: ↑	↑ C18:0, C20:0, C24:1 to Ath and Ob, ↑ C16:0, C23:0 to Ath	-	
	Obese (9 M/5 F)	40 ± 2	VO_2_peak-Rel: 24 ± 3 *	Obese	Serum: Not different in lean	-	-	
Bergman et al. (2016, 2018) [37,74]	Athletes (*n* = 15)	41 ± 1	VO_2_peak-Rel: 48 ± 4 *	Lean	C:24: > T2D and Obese	-	-	Not total, but C:18
	T2D (*n* = 15)	43 ± 1	VO_2_peak-Rel: 19 ± 3 *	Obese-T2D	C:18: > Ath = Obese	-	-	
	Obese (*n* = 14)	40 ± 2	VO_2_peak-Rel: 24 ± 3 *	Obese	-	-	-	
Søgaard et al. (2019) [28]	Young (11 M)	23 ± 1	VO_2_peak-Rel: 46 ± 1 *	Lean	622 ± 74	> C:16, C:18,C:22 vs. both OLD		In young: HOMA-IR correlated with C16:0 and total Cer. No association in the old.
	Young Trained (16 M)	23 ± 1	VO_2_peak-Rel: 53 ± 2 *	Lean	661 ± 91		-	
	Old (18 M)	66 ± 1	VO_2_peak-Rel: 31 ± 1 *	Lean/OW	410 ± 66	-	-	
	Old Trained (15 M)	64 ± 1	VO_2_peak-Rel: 43 ± 4 *	Lean	550 ± 74	-	-	
Perreault et al. (2018) [38]	Lean (8 M/6 F)	43 ± 2	Sed (<2 h/wk PA)	Lean	-	-	-	(−) Many sarcolemmal lipids, Mito/ER, nuclear C18; (+) mito ER/DAGs
	Athletes (10 M/6 F)	43 ± 1	Masters Athletes	Lean	-	-	-	
	Obese (8 M/7 F)	42 ± 2	Sed (<2 h/wk PA)	Obese	-	-	-	
	T2D (7 M/5 F)	46 ± 2	Sed (<2 h/wk PA)	Obese-T2D	> all, most in sarcolemmal	> C16, C18, sarcolemmal, > C18 in nuclear fraction	-	

↑, increase; ↑↑, large increase; Abs, absolute; Cer, ceramides; DAG, diacylglycerol; ER, endoplasmic reticulum; F, female: h, hour; HOMA-IR, homeostatic model assessment of insulin resistance; IGT, impaired glucose tolerance; IMTG, intramuscular trigycerides; IS, insulin sensitivity; M, male; Min, minutes; Mito, mitochondrial; *n*, number of subjects; ng/mg, nanogram per milligram; nmol/g, nanomole per gram; nmoL/mg, nanomole per milligram; NW, normal weight; OW, overweight; PA, physical Activity; RBCs, red blood Cells; Rel, relative; Sat, saturated; Sed, sedentary; T1, myosin heavy-chain 1; T2D, persons with type-2 diabetes mellitus; VO_2_peak, peak oxygen uptake; y, years; μg/mg, micrograms per milligram; Rel, relative * (milliliters per kilogram body weight per minute ); ‡ (milliliters per kilogram of fat free mass per minute).

**Table 4:** Exercise training studies in humans.

Reference	Subjects	Age (y)	Aerobic Fitness	Health/BMI	Training	Muscle Ceramide	Ceramide Species	Muscle Fat or IMTG	Associations
Bruce et al. (2004) [76]	Control (*n* = 6)	46 ± 3	Rel: ~30 *	OW	8 wk, AET	-	-	BL <, ↔ IMTG	No
	T2D (*n* = 7)	48 ± 2		Obese		-	-	BL >, ↓ IMTG	
Bruce et al. (2006) [77]	Obese (4 M/5 F)	36 ± 3	Rel: 24 ± 2 *	Obese	8 wk, AET	BL (734 nmol/g), ↓	C16:0, C16:1, C18:0, C18:1, C18:2, C20:0 ↓	↔	No
Dube et al. (2008) [78]	Old (9 M/16 F)	66.4 ± 0.8	Rel: 34 ± 7 *, ↑ 7%	OW/Obese	16 wk, AET	↓	-	↑ ~21%	Cer (*r*^2^ = 0.46, *p* = 0.01)
Dube et al. (2011) [79]	DIWL (3 M/5 F)	67 ± 2	Rel: 31 ± 2 *, ↔	OW-Obese, NGT-IGT	DIWL	BL=, ↔	↓ C14:0, C20:0, C24:0; ↑C24:1	BL=, ↓ IMTG	BL total and some cer species, ↓ in C16:0 and C24:1 with improved IS
	Ex (4 M/4 F)	68 ± 2	Rel: 32 ± 2 *, ↔	OW-Obese, NGT-IGT	16 wk; AET	↓ 30–40% (>DIWL)	↓ all but C16:0, C18:1 (>DIWL for C18:0, C24:1)	BL=, ↑ IMTG	
Devrives et al. (2013) [80]	Lean (12 F)	41 ± 2	Rel: 26 ± 1 *, ↑	Lean	12 wk, AET	~110 nmol/g dw, ↔	-	AET localizes IMCs close to Mito and IMF, away from SS	-
	Obese (11 F)	40 ± 3	Rel: 19 ± 2 *, ↑	Obese		~130 nmol/g dw, ↔	-		-
Samjoo et al. (2013) [81]	Lean (9 M)	38 ± 3	Rel: 47 ± 2 ‡, ↑	Lean	12 wk, AET	↔	-	AET localizes IMCs close to Mito and IMF, away from SS	No
	Obese (9 M)	39 ± 3	Rel:45 ± 2 ‡, ↑	Obese		~100 nmol/g, ↔	-		
Coen et al. (2015) [82]	Control (*n* = 51)	42.1 ± 9.9	Rel: ~18 ‡, ↑	OW/Obese	None, RYGB	↓	↓ 16,18:1, 24:1	↓↓	No
	Ex (*n* = 50)	41.6 ± 9.3		OW/Obese	post RYGB; 12 wk	↓↓	↓ 16,18,18:1, 24:1	↓	
Kasumov et al. (2015) [83]	NGT (8 M/6 F)	62 ± 2	Absolute: 2 ± 0.1 L/min	Obese	12 wk	Plasma: BL=, ↓	↓ C14:0, C16:0, C24:0	-	Total and C:14 cer negative with GIR change
	T2D (5 M/5 F)	65 ± 2		T2D-Obese		Plasma: BL= ↓	↓ C14:0, C16:0, C18:1, C24:0	-	
Søgaard et al. (2016) [84]	Control (10 M/6 F)	31.3 ± 1.5	Rel: 42 *	OW	10 wk, AET	BL=	No difference at BL, ↓ C22:0	-	No
	Offspring (12 M/7 F)	33.1 ± 1.4	Rel: 38 *	OW-offspring of T2D	10 wk, AET	BL=	No difference at BL, ↓ C22:0	-	
McKenzie et al. (2017) [85]	HipFx (3 M/4 F)	78.4 ± 13.3	Low	OW	12 wk RE and RET	~100 nmol/g, ↔	↔	-	No
Shepherd et al. (2017) [86]	Obese (8 M)	24 ± 2	Rel: 34 *; ↑	Obese	4 wk, HIIT	↓	↓ Cer 18:0	↔	No
	Obese (8 M)	26 ± 2		Obese	4 wk, AET	↓	↓ Cer 18:0	↔	No
Søgaard et al. 2019 [25]	Young (5 M/9 F)	32 ± 2	Rel: ~27*	Obese	6 wk, HIIT	↔	↔	↔	Not reported
	Old (11 M/11 F)	63 ± 1		Obese	6 wk, HIIT	↓	↓ Cer Sat, 18:0	↔	

>, greater than; <, less than; ↑, increase; ↓, decrease; ↓↓, large decrease; ↔, no change; Abs, absolute; AET, aerobic exercise training; BL, baseline; BL=, no difference at baseline between groups; BMI, Body Mass Index; COX, cyclooxygenase; Cer, Ceramide; DIWL, diet-induced weight loss; Ex, exercise; dw, dry tissue weight; GIR, glucose infusion rate; HIIT, high intensity interval training; HipFx, hip fracture patients; IGT, impaired glucose tolerance; IMC, intramuscular ceramides; IMF, intramyofibrillar; IMTG, intramuscular triglycerides; IS, insulin sensitivity; M, men; Mito, mitochondrial; *n*, number of subjects; NGT, normal glucose tolerance; nmol/g, nanomole per gram; OW, overweight; Rel, relative; RET, resistance exercise training; RYGB, Roux-en-Y gastric bypass; SS, subsarcollemal; T2D, persons with type-2 diabetes mellitus; wk, week; Rel, relative * (milliliters per kilogram body weight per minute); ‡ (milliliters per kilogram of fat free mass per minute).

## Supplementary Materials

The following are available online at https://www.mdpi.com/1422-0067/21/4/1514/s1, Table S1: Methods of cross-sectional studies and muscle ceramides in humans, Table S2: Methods of exercise training studies in humans.
