# Supplementary material for: Influence of Exercise Training on Skeletal Muscle Insulin Resistance in Aging: Spotlight on Muscle Ceramides
Source: Int J Mol Sci. 2020 Feb 22;21(4):1514. doi: 10.3390/ijms21041514 (PMC7073171; doi:10.3390/ijms21041514)
Supplement: Supplementary file 1 [file ijms-21-01514-s001.pdf]

Supplementray Tables

Table S1. Methods of chronic exercise training and muscle ceramides in humans.

Reference	Ceramide Method	IS Method	IS	Other
Helge et al. (2004) [65]	TLC, GLC	None<br>None	-<br>-	↓ SMase in TR
Skovbro et al. (2008) [66]	TLC, GLC	2-step HE Clamp (28 and 80 mU m/min), (mg·kg LBM^{-1}·min^{-1})	Low: ~1, High ~5<br>Low: ~3.5, High ~10<br>Low: ~6.5, High ~13<br>Low: ~11, High ~17	-<br>-<br>-<br>-
Amati et al. (2011) [67]	HPLC, MS/MS	HE Clamp (40 mU/m2/min) with stable isotope tracer dilution (mg·kg LBM^{-1}·min^{-1})	6.3 ± 1<br>9.7 ± 1<br>12.3 ± 1	Mito volume density and SDH content > in Athletes
Chow et al. (2014) [68]	UPLC/MS/MS	HE Clamp	68 ± 4 ^<br>49 ± 4 ^	PalC and OC ↑ in Trained at BL
Chow et al. (2017) [69]	-<br>-	HE Clamp (mg·kg FFM^{-1}·min^{-1})	12 ± 1, 13 ± 1<br>9 ± 1, 11 ± 1	-<br>-
Bergman et al. (2010) [70]	-<br>-	IVGTT (Si, mU·l^{-1} min^{-1})	10.9 ± 1.5<br>5.3 ± 0.7	> IMTG FSR, SCD1 mRNA and protein<br>PL: > 16:0,18:2; < 18:0,18:1
Baranowski et al. (2011) [71]	HPLC	-<br>-	-<br>-	S1P and SA1P ↑ Ex recovers in Plasma<br>> S1P in plasma, < SA1P in RBC (S1P is protective)
Bergman et al. (2012) [72]	-<br>-<br>-	IVGTT (Si, mU·l^{-1} min^{-1})	3.2 ± 0.4<br>2.4 ± 0.6<br>12.6 ± 1.7	-<br>-<br>-
Bergman et al. (2015, 2016, 2018) [37,73,74]	LC/ESI/MS/MS	IVGTT (Si, mU·l^{-1} min^{-1})	9.4 ± 1.0<br>2.0 ± 0.3<br>2.93 ± 0.2	-<br>-<br>-
Bergman et al. (2016, 2018) [37,74]	LC/ESI/MS/MS	IVGTT (Si, mU·l^{-1} min^{-1})	9.4 ± 1.0<br>2.0 ± 0.3<br>2.93 ± 0.2	These findings were largely driven by changes in T2D<br>includes a bout of Ex<br>-
Søgaard et al. (2019) [28]	TLC-HPLC	Insulin 120-min OGTT (pmol·L^{-1})	87.9 ± 14.8<br>87.9 ± 14.8<br>97.6 ± 55.3<br>273 ± 65<br>~13	-<br>-<br>-<br>-

Table S1. *Cont.*

Reference	Ceramide Method	IS Method	IS	Other
Perrealt et al. (2018) [38]	LC/ESI/MS/MS	HE Clamp, w/ glucose tracers (mg·kg BW^{-1}·min^{-1})	~9<br>~13<br>~4.5<br>~2	No effect on Cytosolic fractions

↑, increase; ↓, decrease; BL, baseline; ESI, electrospray ionization; Ex, exercise; FFM. fat-free mass; FSR, fractional synthesis rate; GLC, gas–liquid chromatography; HE, hyperinsulinemic euglycemic; HPLC, high performance liquid chromatography; IMTG, intramuscular trigycerides; IS, insulin sensitivity; IVGTT, intravenous glucose tolerance test; LBM, lean body mass; LC, liquid chromatography; min, minutes; Mito, mitochondrial; mg·kg FFM^{-1}·min^{-1}, milliliters per kilogram of fat free mass per minute; MS/MS, tandem mass spectrometry; nSMase, neutral sphingomyelinase; OC, oleoyl-carnitine; PalC, palmitoyl-carnitine; RBCs, red blood Cells; SA1P, sphinganine-1-phosphate; SCD1, stearoyl-CoA desaturase-1; SDH, succinate dehydrogenase; SMase, sphingomyelinase; T2D, persons with type-2 diabetes mellitus; TLC, thin-layer chromatography; TR, Trained; UPLC, ultra performance liquid chromatography; ^ μmol glucose infused·kg FFM^{-1}·min.

Table S2. Methods of Exercise Training Studies in Humans.

Reference	PA Modification	Ceramide Method	IS Method	IS (PRE)	IS (Post)	Note
Bruce et al. (2004) [76]	3 d/wk, 8 wk, AET, cycle	-<br>-	OGTT, HE Clamp (mg·kg $FFM^{-1}·min^{-1}$)	~8<br>~3	~11, ↑ ~30%<br>~5, ↑ ~30%	
Bruce et al. (2006) [77]	5 d/wk, 8 wk, AET, cycle	TLC, GLC	OGTT		↑	↑ CPT1 and Mito FA oxidation
Dube et al. (2008) [78]	4–5 d/wk, 16 wk; AET	HPLC-MS/MS (? Std)	HE Clamp (mg·kg $FFM^{-1}·min^{-1}$)	~8	~9 ↑	
Dube et al. (2011) [79]	DIWL<br>4–5 d/wk, 16 wk; AET	HPLC-MS/MS (? Std)	HE Clamp (40 mU/ml/min) w/ stable isotope tracer dilution (mg·kg $FFM^{-1}·min^{-1}$)	~7.7<br>~7.7	~9.5<br>~9	BL=, ↓ SDH,Glycogen<br>BL=, ↑ SDH,Glycogen
Devrives et al. (2013) [80]	2–3 d/wk, 12 wk, AET, cycle	DKA, TLC	HOMA-IR	1.1<br>2.3	1.2, ↔<br>2.1 ' ↔	↑ mito markers with AET
Samjoo et al. (2013) [81]	2–3 d/wk, 12 wk, AET, cycle	DKA, TLC	OGTT, HOMA-IR	1.97 ± 0.26 (> lean)<br>3.31 ± 0.47	↑<br>↑	
Coen et al. (2015) [82]	None, RYGB<br>1–3 mo post RYGB; 3–5 d/wk for 12 wk	HPLC ESI/MS/MS (? Std)	IVGTT, HOMA-IR (mg·kg $BW^{-1}·min^{-1}$)	2.29 ± 1.43<br>2.27 ± 1.14	3.76 ± 2.19<br>4.52 ± 2.56	↔ sphingolipid bases
Kasumov et al. (2015) [83]	5 d/wk, 12 wk	HPLC ESI/MS/MS (C17, C25 std)	HE Clamp (40 mU/ml/min) (mg·kg $FFM^{-1}·min^{-1}$)	~2.5<br>~1.8	~4, ↑ 71 ± 21%<br>~2.4, ↑ 48 ± 13%	undetectable in some subjects
Søgaard et al (2016) [84]	3 d/wk, 10 wk, AET<br>3 d/wk, 10 wk, AET	LC/ESI/MS/MS (C17 std)	HE Clamp (mg·kg $FFM^{-1}·min^{-1}$)	7.6 ± 0.8<br>5.4 ± 0.4	8.47 ± 0.73 ↑<br>6.27 ± 0.52 ↑	
McKenzie et al. (2017) [85]	12 wk multicomponent RET program	HPLC ESI/MS (C17 std)	-	-	-	
Shepherd et al (2017) [86]	Sprints (4–7 × 30 s at 200% Wmax, 3 d/wk, 4 wk)<br>AET (40–60 min cycling at ~65% VO_2peak, 5 d/wk, 4 wk)	ESI/MS/MS normalized to PC (C17 std)	Matsuda	1.8 ± 0.1<br>1.7 ± 0.1	2.0 ± 0.2, ↑ 11%<br>2.1 ± 0.2, ↑ 24%	↑ COX, IMTG near mito, and mito size, PLIN2 PLIN3 and PLIN5 in Type-1 myofibers, PLIN3 in Type-2
Søgaard et al. (2019) [25]	3 d/wk, 6 wk, HIIT<br>3 d/wk, 6 wk, HIIT	LC/ESI/MS/MS	HOMA-IR	2.14 ± 0.24<br>1.88 ± 0.23	2.31 ± 0.38<br>1.99 ± 0.30	

↑ , increase; ↓, decrease; ↔, no change; AET, aerobic exercise training; BL=, no difference at baseline between groups; COX, cyclooxygenase; CPT1, carnitine palmitoyltransferase I; DIWL, diet-induced weight loss; DKA, diacylglycerol kinase assay; ESI, electrospray ionization; FA, fatty acids; FFM, fat free mass; GLC, gas–liquid chromatography; HE, hyperinsulinemic euglycemic; HIIT, high intensity interval training; HOMA-IR, homeostatic model assessment of insulin resistance; HPLC, high performance liquid chromatography; IMTG, intramuscular triglycerides; IS, insulin sensitivity; IVGTT, Intravenous glucose tolerance test; LC, liquid chromatography; min, minutes; Mito, mitochondrial; mg·kg $FFM^{-1}·min^{-1}$, milliliters per kilogram of fat free mass per minute; MS, mass spectrometry; MS/MS, tandem mass spectrometry;

mU/mL/min, micro units per milliliter per minute; OGTT, oral glucose tolerance test; PLIN, perilipin; RET, resistance exercise training; RYGB, Roux-en-Y gastric bypass; SDH, succinate dehydrogenase; TLC, thin-layer chromatography; Type-1, myosin heavy-chain 1; Type-2, myosin heavy-chain 2; VO_2, volume of oxygen consumed; VO_2peak, peak oxygen uptake; wk, week; Wmax, watts at VO_2max.
